# C3435T Polymorphism of the ABCB1 Gene in Polish Patients with Inflammatory Bowel Disease: A Case–Control and Meta-Analysis Study

**DOI:** 10.3390/genes12091419

**Published:** 2021-09-15

**Authors:** Paweł Petryszyn, Robert Dudkowiak, Agnieszka Gruca, Ewa Jaźwińska-Tarnawska, Paweł Ekk-Cierniakowski, Elżbieta Poniewierka, Anna Wiela-Hojeńska, Krystyna Głowacka

**Affiliations:** 1 Department of Clinical Pharmacology, Wroclaw Medical University, 50-571 Wroclaw, Poland; agnieszkagruca1995@gmail.com (A.G.); tje1@wp.pl (E.J.-T.); anna.wiela@gmail.com (A.W.-H.); kryglowacka@gmail.com (K.G.); 2 Department of Gastroenterology and Hepatology, Wroclaw Medical University, 50-571 Wroclaw, Poland; robindud@op.pl (R.D.); elzbieta.poniewierka@gmail.com (E.P.); 3 Warsaw School of Economics, 00-968 Warsaw, Poland; pawel.ekk.cierniakowski@gmail.com

**Keywords:** Crohn’s disease, ulcerative colitis, P-glycoprotein, association, meta-analysis, geographical variations, functional genomics, pharmacogenetics

## Abstract

P-glycoprotein encoded by the ABCB1 gene constitutes a molecular barrier in the small and large bowel epithelium, and its different expression may influence susceptibility to inflammatory bowel disease (IBD). We aimed to assess the contribution of the C3435T polymorphism to disease risk in the Polish population. A total of 100 patients (50 Crohn’s disease (CD), 50 ulcerative colitis (UC)) and 100 healthy controls were genotyped for the single nucleotide polymorphism (SNP) C3435T by using the PCR-RFLP method. Patients were classified on the basis of disease phenotype and the specific treatment used. A meta-analysis was carried out of our results and those from previously published Polish studies. There was no significant difference in allele and genotype frequencies in IBD patients compared with controls. For CD patients, a lower frequency of TT genotype in those with colonic disease, a lower frequency of T allele, and a higher frequency of C allele in those with luminal disease were observed, whereas for UC patients, a lower frequency of CT genotype was observed in those with left-sided colitis. A meta-analysis showed a tendency towards higher prevalence of CC genotype in UC cases. These results indicate that the C3435T variants may confer a risk for UC and influence disease behaviour.

## 1. Introduction

Inflammatory bowel diseases (IBD) are heterogeneous disorders that cause chronic inflammation of the gastrointestinal tract with possible extraintestinal manifestations. Two main subtypes, i.e., Crohn’s disease (CD) and ulcerative colitis (UC), can be distinguished [1]. They have many clinical presentations and pathological characteristics. The most typical for CD include diarrhoea, abdominal pain and systemic features like anorexia, malaise and fever. UC symptoms are most often bloody diarrhoea, colicky abdominal pain and tenesmus. In CD the inflammation can affect any part of the gastrointestinal tract from the mouth to the anus, and may trigger fibrosis or fistula formation. In UC it is confined to the colonic mucosa (with distortion of crypt architecture, crypt abscesses and basal plasmacytosis) and epithelium (with mucin depletion, and Paneth cell metaplasia) [2,3]. IBD can occur in different geographic regions; in a systematic review of population-based studies the highest reported prevalence values were in Europe and North America (322 per 100,000 in Germany and 319 per 100,000 in Canada for CD and 505 per 100,000 in Norway and 286 per 100,000 in the US for UC, respectively) [4]. In a large multicentre study conducted between 1991 and 1993 in 12 European countries, the CD and UC incidence were estimated at 5.6 and 10.4 per 100,000 person-years, respectively [5]. Currently, the Western European countries, North America and Australia are characterized by a stable incidence and rapidly increasing prevalence due to the low mortality of IBD patients. In 2030 it will reach about 1% in many regions [6].

The pathogenesis of IBD remains not fully understood, but may require an interaction between genetic predisposition and combination of persistent bacterial infection, defective mucous barrier and imbalance in the regulation of the immune system [2]. Genetic factors play an important role in susceptibility to IBD, as has been reflected in twin studies [7,8]. The NOD2/CARD15 gene (nucleotide-binding oligomerization domain, caspase recruitment domain), located on chromosome 16, was the first identified gene related to CD [9,10]. Wild-type protein NOD2 activates the nuclear factor kappa B, thanks to which it reacts to bacterial lipopolysaccharides. There is a deficiency in such induction in patients with the mutant form of NOD2 [11]. Three mutations in the NOD2/CARD15 gene, i.e., Arg702Trp, Gly908Arg and Leu1007fsinsC have been associated with ileal involvement, structuring complications and earlier disease onset [12]. Recently, the link between mutations in the NOD2/CARD15 gene and low anti-TNFα through levels in CD patients has been reported [13,14]. Nevertheless, genetics explains only a small portion of disease variance and has little ability to predict relapses and response to treatment [15]. Environmental factors may modify both the risk of IBD and its course. Smoking is a risk factor for CD, but may lower the risk of developing UC [16,17]. Patients with incident IBD are more likely than matched controls to have a history of gastroenteritis [18]. Non-steroidal anti-inflammatory drugs increase the risk of CD and UC, but the effect is relatively small [19]. Alterations of the epigenome may link genetic susceptibility and environmental influences. DNA methylation changes in gene promoters have been found to be functionally involved in the regulation of gene expression in patients with IBD [20,21,22].

P-glycoprotein (P-gp) is one of the most extensively studied human adenosine triphosphate (ATP)-binding membrane cassette transporters and is encoded by the multidrug resistance ABCB1 gene (previously known as MDR1). P-gp catalyses the translocation of its substrates against a concentration gradient by using the energy generated from the hydrolysis of ATP. It prevents uptake of drugs and toxins into the blood capillaries and limits the bioavailability of many orally administered drugs [23]. In addition, P-gp is possibly involved in the complex interaction between bacteria and the host organism [24]. P-gp is expressed in many human tissues, including peripheral blood lymphocytes, epithelial cells in the small and large intestine, hepatocytes (at their biliary pole), pancreatic ductile cells, proximal tubular cells in kidneys, the epithelium of the brain choroids plexus, the capillaries of the brain, placenta, etc. [25,26]. The ABCB1 gene, encoding the P-glycoprotein, is located on chromosome 7q21.1 and consists of 28 introns and 28 exons. It shows high polymorphism; so far, 50 single nucleotide polymorphisms (SNPs) have been described. The most frequently studied polymorphisms of the ABCB1 gene are C1236T (rs1128503), G2677T/A (rs2032582) and C3435T (rs1045642) [24,27,28]. Hoffmeyer et al. were the first to find a 2-fold reduction in P-gp expression in duodenal biopsies in healthy Caucasian volunteers homozygous for the mutant 3435T allele in exon 26. They showed an increased plasma digoxin concentration after oral administration compared to subjects homozygous for the C3435 allele (wild type) [29].

The ABCB1 gene is an attractive candidate for the pathogenesis of IBD and perhaps response to therapy. Its role in IBD was investigated in a mouse knockout model (mdr1a-/-). These mice, previously healthy, spontaneously, even if maintained under specific pathogen-free conditions, developed colitis, which resembles UC in humans. The colitis was prevented and reversed by the administration of antibiotics, suggesting that the intestinal flora is necessary to initiate and perpetuate the inflammation [30]. Nevertheless, a limited number of studies analysing C3435T SNP in IBD has yielded conflicting results [31]. Two meta-analyses confirmed an association between C3435T and UC, but not with CD [32,33]. Interestingly, some drugs crucial in the therapy of IBD are also P-gp substrates like glucocorticoids, cyclosporine and methotrexate [33]. P-gp expression was significantly elevated in peripheral blood lymphocytes both in CD patients requiring bowel resection and UC patients requiring proctocolectomy for failed medical therapy [34].

Therefore, we aimed to investigate the contribution of ABCB1 C3435T polymorphism to IBD susceptibility, the relationship between genotype and disease phenotype, as well as drug exposure, in a cohort of well clinically characterized patients originating from the Lower Silesia region in Poland, and to embed our association study results in the context of previous studies carried out in the Polish population.

## 2. Materials and Methods

### 2.1. Patients and Controls

The study had a case–control design. The cases consisted of 100 patients, age 19–81 years, with IBD, recruited consecutively from the Department of Gastroenterology and Hepatology of Wroclaw Medical University, Poland in the period from September 2019 to December 2020. The diagnoses of CD and UC were made by a gastroenterologist based on endoscopic, radiological, and/or histopathological examinations in compliance with established clinical guidelines and criteria [35,36]. One hundred healthy controls, age 18–60 years, were recruited from the Lower Silesia region to match the recruitment area.

### 2.2. Genotyping

The genomic DNA samples of patients and controls were extracted from peripheral blood leucocytes with the use of E.Z.N.A.^®^ Blood DNA Mini Kit (Omega Bio-tek, GA, Norcross, US) according to the procedure provided by the manufacturer at the Department of Clinical Pharmacology of Wroclaw Medical University, Poland.

The determination of C3435T polymorphism in exon 26 of the ABCB1 gene was performed with the modified method of Siegmund and collaborators using Polymerase Chain Reaction–Restriction Fragment Length Polymorphism (PCR-RFLP) [37]. The PCR product was digested by the restriction enzyme MboI (EURx, Gdansk, Poland). Three allelic variants of functional C3435T polymorphism could be identified: homozygous wild type (3435CC), heterozygous (3435CT) and homozygous (3435TT) mutant types. Two restriction fragments (158 bp and 39 bp) were detected from the C3435 allele. The 3435T allele was not digested and produced a long band at 197 bp. The accuracy of genotyping was confirmed by direct sequencing of 15 random samples.

### 2.3. Phenotypic Assessment

A thorough analysis of medical records was performed, and detailed phenotypic information was available for all patients from the study group. For both CD and UC, the Montreal classification was employed [38]. For CD, location was determined by the maximum extent of disease involvement at any time before the first surgery and disease behaviour was labelled by current or past behaviour subtypes. Inflammatory disease was diagnosed in the absence of stenotic or perforating behaviour. For UC, severe disease was consistent with the presence of at least 6 stools/day, severe rectal bleeding together with signs of systemic inflammation (tachycardia > 90 bpm, temperature > 37.5 °C, haemoglobin < 10.5 g/dL, C-reactive protein > 30 mg/L). Other clinical data we searched for were: age at diagnosis, duration of follow-up, previous abdominal surgery (either bowel resection in CD or colectomy in UC). Particular attention was paid to medical therapy and, more specifically, we accounted for the use of 5-ASA, systemic corticosteroids, immunosuppressant therapy (azathioprine, 6-mercaptopurine, methotrexate) and anti-TNFs (infliximab, adalimumab).

### 2.4. Meta-Analysis

In an attempt to evaluate the potential association of C3435T polymorphism of ABCB1 with IBD in a wider context, we carried out a meta-analysis of studies on Polish population. We performed systematic search of PubMed and EMBASE databases for articles published until 27th of March 2021. Our search strategy was as follows: (‘multidrug resistance protein 1/exp OR ‘multidrug resistance protein 1’ OR ‘abcb1 protein’:ab,ti OR ‘mdr1’:ab,ti OR ‘p glycoprotein’:ab,ti) AND (‘inflammatory bowel disease’/exp OR ‘inflammatory bowel disease*’:ab,ti OR ‘crohn disease’/exp OR ‘ulcerative colitis’/exp) AND (‘polish’/exp OR ‘polish’:ab,ti OR ‘poland’:ab,ti,ad,ff,ca) AND ([english]/lim OR [polish]/lim) AND [humans]/lim AND ([embase]/lim OR [medline]/lim). A total of 11 records were identified. A detailed review abstracts and full texts enabled inclusion of two studies. Three studies were excluded because of inadequate population (colorectal cancer, peptic ulcer disease, children), in one study different ABCB1 polymorphism (i.e., C1236T) was analysed, another one was not a gene-association but gene-expression study, and four studies were review articles. Characteristics of eligible studies with the allele and genotype frequencies are shown in Table 1. In both of them, the C3435T polymorphism was assessed by using the PCR-RFLP method.

After these results were pooled with our own data, a total of 124 CD cases, 136 UC cases and 338 controls were included in the meta-analysis.

### 2.5. Statistical Analysis

The study was of case–control design. The distribution of individual characteristics was evaluated by simple descriptive statistics. Values were presented as mean ± SD. To determine whether the observed allele frequency distribution remained in the genetic equilibrium, the Hardy–Weinberg equation was tested via the Court lab-HW Calculator.xls.

Allele and genotype frequencies were compared between cases and controls, as well as between specific phenotypic disease subgroups (of at least 10 patients) and controls, through the use of chi-square tests. Odds ratios (ORs) were given with 95% confidence intervals (CIs) and 2-sided *p*-values. *p*-values were reported uncorrected for multiple testing and those <0.05 were considered statistically significant.

In meta-analysis for any individual study, ORs and 95% Cis were calculated and T and C allele as well as CC, CT and TT genotype frequencies were compared across studies. Statistical heterogeneity was evaluated with Cochran’s Q-statistic and quantified with the I^2^ statistic. In all comparisons, the hypothesis about the homogeneity of the studies could not be rejected (*p* > 0.05 and I^2^ < 50%); however, both fixed- and random-effects models were employed. Computations were made using the R-package version 4.0.3 software and the “meta” package version 4.19.0 for meta-analysis purposes.

## 3. Results

### 3.1. Demographics and Clinical Characteristics

A total of 50 CD cases, 50 UC cases, and 100 healthy controls were genotyped for C3435T. The proportion of males was higher in UC (64%) than CD (50%). The mean age at diagnosis for CD and UC patients was 27.6 and 29.3 years, with a mean duration of follow-up of 8.2 and 9.2 years, respectively. Detailed phenotypic information was available on all IBD cases. The proportions of CD patients with different disease location and behaviour, of UC patients with different disease extent and having severe disease, and of IBD patients as a whole requiring 5-ASA, immunosuppressants, corticosteroids and anti-TNFs, as well as of those who have undergone IBD surgery, are shown in Table 2.

### 3.2. Effect of ABCB1 C3435T Polymorphism on Disease Susceptibility

Control and IBD case genotypes were in Hardy-Weinberg equilibrium. No statistically significant differences were found between either CD or UC patients and the control group for the frequency distribution of T and C alleles as well as of CC, CT and TT genotypes (Table 3).

### 3.3. Genotype–Phenotype Analysis

Frequencies of the C3435T variants in the different IBD phenotypic subgroups were compared with their frequencies in the control group. In the group of CD patients there was a lower frequency of TT genotype (OR = 0; 95% CI 0–1.01) in those with colonic location, a higher frequency of allele C (OR = 2.9; 95% CI 1–9.58) and a lower frequency of allele T (OR = 0.35; 95% CI 0.1–1) in those with only luminal disease, as well as a trend toward a higher frequency of allele C (OR = 2.48; 95% CI 0.95–7.03) and a lower frequency of allele T (OR = 0.4; 95% CI 0.14–1.05) in those with colonic location (Table 4).

In the group of UC patients, there was a lower frequency of CT genotype (OR = 0.18; 95% CI 0.03–0.73) in those with left-sided colitis, together with a trend toward a higher frequency of allele C (OR = 2.35; 95% CI 0.94–6.28) and CC genotype (OR = 3.24; 95% CI 0.74–13.03) and a lower frequency of allele T (OR = 0.43; 95% CI 0.16–1.07) in those with severe disease (Table 5).

### 3.4. Meta-Analysis

**Figure 1:**
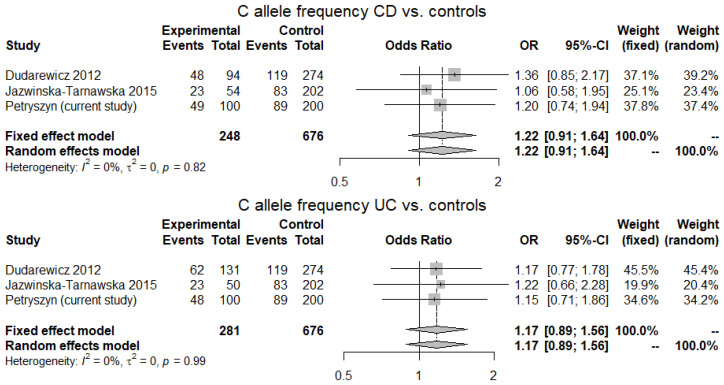
C allele frequency in CD and UC patients vs. controls. Three studies examining an association of C3435T polymorphism of ABCB1 gene with IBD in the Polish population with a total of 124 CD cases, 136 UC cases and 338 controls were included. Heterogeneity was low at I^2^ = 0% (*p* > 0.05). A study-specific OR was >1 for the C3435 allele both for CD and UC in all of the three studies; however, pooled ORs calculated for fixed- and random-effects models were not significant.

**Figure 2:**
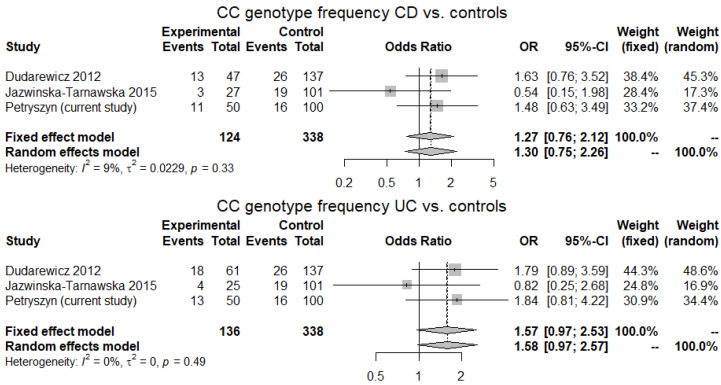
CC genotype frequency in CD and UC patients vs. controls. Three studies examining an association of C3435T polymorphism of ABCB1 gene with IBD in the Polish population with a total of 124 CD cases, 136 UC cases and 338 controls were included. Heterogeneity was low at I^2^ = 0–9% (*p* > 0.05). Two studies produced a study-specific OR > 1 for CC genotype both for CD and UC and the pooled OR for CC genotype in UC tended to be significant (1.57, 95% CI 0.97–2.53; *p* = 0.06 and 1.58, 95% CI 0.97–2.57; *p* = 0.06, for fixed- and random-effects models, respectively).

Each of the three studies included produced a study-specific OR > 1 for the C3435 allele both for CD and UC, although pooled ORs were not significant. A study-specific OR > 1 for CC genotype (both CD and UC) was noted in two studies and the pooled OR for CC genotype in UC tended to be statistically significant (1.58, 95% CI 0.97–2.57; *p* = 0.06, for random-effects model) (Figure 1 and Figure 2).

## 4. Discussion

Inflammatory bowel disease is considered a complex polygenic trait with several genes involved. Linkage analysis, the candidate-gene approach, and genome-wide association studies (GWAS) have all been used to identify IBD susceptibility genes [41,42]. On the basis of multiple GWAS, their meta-analyses, and the use of targeted genotyping array techniques, the number of identified IBD-associated loci has now exceeded 200 [43]. Differences in allele frequencies and effect sizes across populations have been found [44]. These genes can be grouped into biological pathways, i.e., barrier integrity, bacterial recognition and autophagy and genetic alteration within such a wide variety of processes can lead to an increased risk of IBD. It has been suggested that an individual disease phenotype might be determined by the interaction of both environmental factors (external triggers of injury, intestinal microbiome) and genetic heterogeneity [45].

Based on the assumption that interindividual variability of P-gp expression in intestines is linked to the ABCB1 C3435T SNP, attempts have been made to explain whether TT genotype, associated with low intestinal P-gp expression, can predispose to IBD. In the initial case–control study by Schwab et al. (Germany), an increase in the frequency of T allele and TT genotype was confirmed in 149 UC patients, but not in CD patients (*p* = 0.049, OR = 1.4; *p* = 0.005, OR = 2.1, respectively) [46]. This finding was replicated by Ho et al. (Scotland), who reported a statistically significant association between UC and a higher frequency of the mutant T allele (*p* = 0.02, OR = 1.28) and the TT genotype (*p* = 0.04, OR = 1.60) [47], as well as by Brinar et al. (Croatia) who found a higher frequency of the TT genotype in UC patients (*p* = 0.02, OR = 2.12) [48]. Similarly, a higher risk of UC development for the 3435T allele carriers and the 3435TT homozygotes was observed in the Iranian population [49].

In this study, carried out in an IBD population originating from the Lower Silesia region in Poland, we were not able to corroborate the association between the C3435T polymorphism of ABCB1 gene and IBD. The frequency distribution of T and C alleles and CC, TT and CT genotypes was not different between CD and UC patients and the control group. Our results are in line with those of large studies coming from Germany and Great Britain [50], North America [51], Slovenia [52] and Italy [53], none of them replicating the initially found association of T allele and TT genotype with UC.

Divergent results of the above-mentioned studies may be driven by population heterogeneity, sample size, selection of the control population and lack of statistical power to detect small or moderate effect size. A significant variation of the C3435T allele frequency in different populations has been proven. For example, the frequency of the C3435 allele has been reported as 43–54% in Caucasians, 34–63% in Asians and 73–90% in Africans [54]. Glas et al., using two separate control groups, found a significant association of T allele and UC only when control group 2 (minor allele frequency = 43.7%) was used [55]. Therefore, we sought to embed our results in the context of all of the published Polish studies through a meta-analysis. Interestingly, all included studies produced a study-specific OR > 1 for C allele both for CD and UC (not significant), while CC genotype tended to be significantly more frequent in UC cases compared with controls (OR = 1.58, 95% CI 0.97–2.57; *p* = 0.06). This is in contradiction with the previously cited studies, which either found that T allele or TT genotype may increase the risk of UC, or did not confirm a significant influence of the C3435T polymorphism on the risk of IBD in general. A summary effect size was assessed in two meta-analyses from 2006; Annese et al. found a significant association of 3435T allele and 3435TT genotype with UC (OR = 1.17, *p* = 0.003 and OR = 1.36, *p* = 0.017, respectively) and no association with CD [33], and Onnie et al. reported a significant association of the 3435T allele with UC (OR = 1.12, *p* = 0.013) but not with CD [32]. Urcelay et al. (Spain) found a significant association between the wild CC3435 genotype and CD (*p* = 0.007), recognizing the 3435C allele as a risk factor for CD [56].

The results of our study, although we did not obtain statistical significance, seem to question the current state of knowledge, i.e., that a reduced intestinal barrier function initially linked to low P-gp expression and 3435TT genotype increases the risk of developing UC. They should be approached with great caution. First, our sample was relatively small, as was the total number of Polish cases included in the meta-analysis. Secondly, the effect size of an association between C3435T and UC is rather modest (estimated previously at 12–17%) [32,33].

Of note, 3435C→T mutation in exon 26 is a synonymous SNP (i.e., the amino acid encoded is not altered). There is a linkage disequilibrium between SNPs in exon 26 (C3435T) and exon 21 (G2677T/A), suggesting that initially attributed to the exon 26 SNP different P-gp expression, may result from the associated tri-allelic polymorphism in exon 21. The latter is a non-synonymous SNP (i.e., causes amino acid change, Ala893Ser/Thr) [57]. A synonymous SNP in exon 12 (C1236T) has also been shown to be associated with the C3435T and the G2677T A SNPs [58]. The advantage of haplotype analysis in relation to the role of ABCB1 gene in IBD pathogenesis has been confirmed by Potocnik et al. They found that haplotype defined by the T–T–T alleles (1236T–2677T–3435T) is significantly associated with a higher risk of developing refractory CD (OR = 3.1, *p* = 0.04) and UC (OR = 1.6, *p* = 0.03), what was not true for each of these SNPs analysed separately [52]. Last but not least, TT genotype was reported to be associated with a higher level of ABCB1 mRNA in the duodenum in Japanese healthy subjects what explained lower digoxin serum concentration after administration of a single oral dose to those harbouring the mutant 3435T allele [59], which was in striking contradiction to the results of an original experiment by Hoffmeyer et al. [29]. It is evident that the functional role of C3435T polymorphism varies among studies and further in vivo and ex vivo research is still needed.

To investigate the possibility that C3435T associations were limited to specific disease phenotypes, we classified our cases into clinically well-defined subgroups. In the group of CD patients, we found a lower frequency of TT genotype in those with colonic disease (L2), a lower frequency of T allele, and a higher frequency of C allele in those with luminal disease (B1). In the group of UC patients we found a lower frequency of CT genotype in those with left-sided colitis (E2). No association was seen of C3435T with groups distinguished as per the pharmacotherapy used or the need for surgery. Once again it is necessary to underline that our results should be regarded with caution, given the small sample size, particularly when it comes to subgroup analysis.

In a genotype–phenotype analysis by Ho et al., the risk of UC with the proximally spreading lesions was estimated as 1.70 (p = 0.009) and 2.64 (p = 0.003) for the 3435T allele and the 3435TT genotype, respectively [47]. Ardizzone et al. found T allele to confer a threefold increased risk for developing CD with ileo-colonic localization as compared with the wild-type C one [60]. CC genotype was more frequent in CD patients who were corticosteroid dependent than in those responsive to corticosteroids [61]. There was a higher frequency of 3435TT genotype in CD patients who did not respond to azathioprine [62]. However, any impact of C3435T polymorphism with respect to response to the therapy was observed in 594 patients using systemic steroids and 297 patients taking immunosuppressants [53].

It is very difficult to interpret these results, which are so divergent, and associate specific C3435T alleles or genotypes with a specific disease phenotype or response/non-response to treatment. However, due to the heterogeneity of inflammatory bowel disease and the fact that sometimes it can hardly be possible to predict the course of the disease in a given patient, it is always worth looking for the relationship between the phenotype and genetic factors. We acknowledge several limitations of our study: the sample was relatively small; we tested only one ABCB1 polymorphism, and therefore haplotype analysis was not possible; we did not examine the expression of the ABCB1 gene in the duodenum; and we did not correlate that expression with the expression of, e.g., CYP3A4, another known molecular barrier component in the small intestinal epithelium.

The strengths of our study are the association of C3435T variants with different IBD phenotypes and treatment response (revised for the first time in the Polish population) and the embedding the results of current case–control study in the context of other studies in the Polish population. An intriguing conclusion is the predominance of C allele in patients with both CD and UC (statistically insignificant), as well as the predominance of CC genotype in UC patients, striving for statistical significance. An absolutely principal question that arises here relates to the level of expression of the ABCB1 gene with respect to its variants (considering opposite results yielded by German and Japanese authors), and whether this level shows differences depending on the studied population (ethnicity, health vs. disease, etc.). Indeed, in order to dispel some of the uncertainties that arose in the course of the current study, we have already planned its continuation with another SNPs (C1236T and G2677T/A) in addition to C3435T being assessed, enterocolonic biopsies being taken and tissue P-gp expression being measured.

## Figures and Tables

**Table 1:** Distribution of C3435T alleles and genotypes (cases and controls) in included studies.

Studies		Allele		Genotype		
		C	T	CC	CT	TT
Jazwinska-Tarnawska 2015 [39]	CD = 27	23	31	3	17	7
	UC = 25	23	27	4	15	6
	Controls = 101	83	119	19	45	37
Dudarewicz 2012 [40]	CD = 47	48	46	13	22	12
	UC = 61	62	60	18	26	17
	Controls = 137	119	155	26	67	44

**Table 2:** Demographics and clinical characteristics of 100 IBD patients and 100 controls.

Characteristics		CD	UC	Controls
Sex (M/F) (%)		25/25 (50/50)	32/18 (64/36)	45/55 (45/55)
Mean age (±SD)		35.8 (±13.7)	38.5 (±14.9)	37.2 (±12.5)
Mean age at diagnosis (±SD)		27.6 (±13.8)	29.3 (±13.1)	
Disease location (CD)	L1 (ileal)	12		
	L2 (colonic)	12		
	L3 (ileocolonic)	26		
Disease behaviour (CD)	B1 (inflammatory)	10		
	B2 (stricturing)	35		
	B3 (penetrating)	15		
Disease extent (UC)	E1 (ulcerative proctitis)		2	
	E2 (left-sided colitis)		16	
	E3 (pancolitis)		32	
Severe disease (UC)			13	
5-ASA		47	48	
Immunosuppresant therapy		31	22	
Corticosteroid use		31	35	
Anti-TNF		12	5	
IBD surgery		17	7	

**Table 3:** Distribution of C3435T alleles and genotypes (cases and controls).

Subjects	Allele				Genotype					
	C	OR (95% CI)	T	OR (95% CI)	CC	OR (95% CI)	CT	OR (95% CI)	TT	OR (95% CI)
CD = 50	49	1.2 (0.72–1.99)	51	0.84 (0.5–1.39)	11	1.48 (0.56–3.76)	27	0.92 (0.44–1.94)	12	0.81 (0.34–1.88)
UC = 50	48	1.15 (0.69–1.92)	52	0.87 (0.52–1.45)	13	1.84 (0.73–4.55)	22	0.62 (0.29–1.29)	15	1.1 (0.48–2.46)
Controls = 100	89		111		16		56		28	

**Table 4:** Genotype–Phenotype Analysis for C3435T SNP in CD.

Clinical Characteristics	Allele				Genotype					
	C	OR (95% CI)	T	OR (95% CI)	CC	OR (95% CI)	CT	OR (95% CI)	TT	OR (95% CI)
Age at diagnosis ≤ 25 years = 33	33	1.25 (0.69–2.26)	33	0.8(0.44–1.46)	7	1.41 (0.44–4.12)	19	1.07 (0.45–2.58)	7	0.69 (0.23–1.89)
Years since diagnosis ≥ 8 years = 23	21	1.05 (0.52–2.09)	25	0.95 (0.48–1.92)	5	1.45 (0.37–4.9)	11	0.72 (0.26–1.98)	7	1.12 (0.35–3.28)
Ileal disease (L1) = 12	12	1.25 (0.49–3.2)	12	0.8 (0.31–2.06)	4	2.6 (0.51–11.2)	4	0.4 (0.08–1.59)	4	*p* = 0.7396, 1.28 (95% CI: 0.26–5.26)
Colonic disease (L2) = 12	16	2.48 (0.95–7.03) ^a^	8	0.4 (0.14–1.05) ^a^	4	2.6 (0.51–11.2)	8	1.57 (0.39–7.58)	0	0 (0–1.01) ^b^
Ileocolonic disease (L3) = 26	21	0.85 (0.43–1.64)	31	1.18 (0.61–2.33)	3	0.69 (0.12–2.71)	15	1.07 (0.41–2.86)	8	1.14 (0.38–3.16)
Inflammatory disease (B1) = 10	14	2.9 (1–9.58) ^b^	6	0.35 (0.1–1) ^b^	4	3.45 (0.64–16.54)	6	1.18 (0.26–6.03)	0	0 (0–1.25)
Stricturing disease (B2) = 35	31	0.99 (0.55–1.78)	39	1.01 (0.56–1.82)	6	1.09 (0.32–3.28)	19	0.93 (0.4–2.19)	10	1.03 (0.39–2.58)
Penetrating disease (B3) = 15	10	0.62 (0.25–1.48)	20	1.6 (0.67–4.03)	1	0.38 (0.01–2.84)	8	0.9 (0.26–3.16)	6	1.71 (0.46–5.96)
Immunosuppresant therapy = 31	28	1.03 (0.55–1.89)	34	0.97 (0.53–1.8)	5	1.01 (0.26–3.26)	18	1.09 (0.45–2.7)	8	0.9 (0.31–2.39)
Corticosteroid use = 31	31	1.25 (0.68–2.3)	31	0.8 (0.44–1.48)	6	1.26 (0.36–3.85)	19	1.24 (0.51–3.13)	6	0.62 (0.19–1.77)
Anti-TNF = 12	9	0.75 (0.28–1.93)	15	1.33 (0.52–3.63)	2	1.05 (0.1–5.66)	5	0.56 (0.13–2.23)	5	1.83 (0.42–7.34)
IBD surgery = 17	11	0.6 (0.25–1.36)	23	1.67 (0.74–4.02)	1	0.33 (0.01–2.44)	9	0.88 (0.28–2.87)	7	1.79 (0.52–5.82)

^a^ *p* = 0.05; ^b^ *p* < 0.05.

**Table 5:** Genotype–phenotype analysis for C3435T SNP in UC.

Clinical Characteristics	Allele				Genotype					
	C		T		CC		CT		TT	
Age at diagnosis ≤ 25 years = 25	18	0.7 (0.35–1.39)	32	1.42 (0.72–2.88)	4	1 (0.22–3.56)	10	0.53 (0.19–1.39)	11	2.01 (0.73–5.44)
Years since diagnosis ≥ 8 years = 26	23	0.99 (0.51–1.91)	29	1.01 (0.52–1.97)	5	1.25 (0.32–4.12)	13	0.79 (0.3–2.05)	8	1.14 (0.38–3.16)
Left-sided colitis (E2) = 16	15	1.1 (0.48–2.49)	17	0.91 (0.4–2.07)	6	3.11 (0.81–11.15)	3	0.18 (0.03–0.73) ^a^	7	1.99 (0.57–6.68)
Pancolitis (E3) = 32	32	1.25 (0.68–2.28)	32	0.8 (0.44–1.47)	7	1.47 (95% CI: 0.46–4.3)	18	1.01 (0.42–2.46)	7	0.72 (0.24–1.97)
Severe disease = 13	17	2.35 (0.94–6.28) ^b^	9	0.43 (0.16–1.07) ^b^	5	3.24 (0.74–13.03) ^b^	7	0.92 (0.24–3.56)	1	0.22 (0–1.59)
Immunosuppresant therapy = 22	20	1.04 (0.51–2.11)	24	0.96 (0.47–1.97)	5	1.54 (0.39–5.22)	10	0.66 (0.23–1.83)	7	1.2 (0.37–3.54)
Corticosteroid use = 35	35	1.25 (0.7–2.23)	35	0.8 (0.45–1.44)	11	2.39 (0.88–6.36)	13	0.47 (0.19–1.09)	11	1.18 (0.46–2.9)

^a^ *p* < 0.05; ^b^ *p* = 0.06.

## Data Availability

The data presented in this study are available on request from the corresponding author.
